# Clopidogrel Loading Dose 300 vs. 600 mg in Patients Undergoing One-Stop Hybrid Coronary Revascularization: A Prospective Single-Center Randomized Pilot Study

**DOI:** 10.3389/fsurg.2021.768860

**Published:** 2021-10-15

**Authors:** Yulin Guo, Dongjie Li, Yingdi Gao, Jing Zhao, Xiangguang An, Yan Liu, Song Gu, Xitao Zhang, Jie Gao, Pixiong Su

**Affiliations:** Department of Cardiac Surgery, Heart Center and Beijing Key Laboratory of Hypertension, Beijing Chaoyang Hospital, Capital Medical University, Beijing, China

**Keywords:** hybrid coronary revascularization (HCR), clopidogrel, major bleeding, pilot study, heart surgery

## Abstract

**Background:** The optimal loading dose of clopidogrel in one-stop hybrid coronary revascularization (HCR) remains an “evidence-free” zone. This study aimed to compare the major bleeding and ischemic thrombotic events between different clopidogrel loading doses (300 vs. 600 mg) in one-stop HCR.

**Methods:** In this prospective, single-center, randomized, and parallel pilot study, 100 patients receiving one-stop HCR were randomly assigned to the clopidogrel loading dose 300-mg group or 600-mg group in a 1:1 ratio. Major bleeding events and composite in-hospital ischemic thrombotic and adverse complications were evaluated after the procedure.

**Results:** The results showed that postoperative mean chest drainage of the first 4 days and total drainage were comparable between the two groups. No differences were found in Bleeding Academic Research Consortium (BARC) coronary artery bypass grafting (CABG) related bleeding (4 vs. 2%, *P* = 1), PLATelet inhibition and patient Outcomes (PLATO) life-threatening bleeding (20 vs. 26%, *P* = 0.48), and PLATO major bleeding (70 vs. 76%, *P* = 0.5) in the two groups. The composite ischemic thrombotic and adverse events were also similar.

**Conclusions:** In patients receiving one-stop HCR, clopidogrel 600 mg loading dose did not increase major bleeding events compared with 300 mg. More sufficient data is necessary to evaluate the potential benefits of 600 mg loading dose in one-stop HCR.

## Introduction

Hybrid coronary revascularization (HCR) was first introduced by Angelini in 1996 (1). It consists of two different revascularization procedures. First, a left internal mammary artery (LIMA) to left anterior descending artery (LAD) anastomosis *via* minimally invasive coronary artery bypass. Second, stents implantation for non-LAD lesions by the percutaneous coronary intervention (PCI) (2). These two separate procedures can be performed simultaneously in a hybrid operating room (one-stop HCR) or within hours, days, or even weeks of each other in the conventional operating theater and catheterization room (staged HCR).

Major bleeding events impair outcomes after cardiac surgery (3), and early stent thrombosis is a serious adverse complication after PCI (4). In one-stop HCR, it is a challenge to balance the bleeding risk of loading dose antiplatelet administration at end of surgical procedure and the risk of acute stent thrombosis owing to the inflammatory response to surgery (5). Routinely, a single loading dose of crushed and dissolved clopidogrel 300–600 mg is administered *via* gastric tube either when the LIMA-LAD graft is completed, before the closure of the thorax, or immediately post-PCI (6–8).

However, in some studies, the exact dose of clopidogrel during one-stop HCR is not clearly described, which highlights the need for more robust clinical guidance. Hence, the optimal loading dose of clopidogrel in one-stop HCR remains an “evidence-free” zone.

The present prospective, single-center, and randomized pilot study aimed to compare the major bleeding and ischemic thrombotic events between different clopidogrel loading doses (300 vs. 600 mg), to investigate the optimal loading dose of clopidogrel in one-stop HCR.

## Methods

### Study Design

The present trial is a prospective, single-center, randomized, and parallel pilot study. All consecutive patients confirmed multivessel disease (MVD) by angiography involving the LAD and a significant lesion in at least one major non-LAD epicardial vessel (> 70%) were screened by the local heart team (consist of an interventional cardiologist, cardiac surgeon, and anesthesiologist). Patients were eligible if they met the inclusion and exclusion criteria after evaluation by the heart team. The inclusion and exclusion criteria are as follows. Inclusion exclusion criteria: age ≥ 18 years old; confirmed MVD by angiography involving LAD (> 70% lesion in at least); indication for revascularization; LAD lesion suitable for surgical revascularization; non-LAD lesions amenable for PCI; patient signed informed consent. Exclusion criteria: acute ST-elevation myocardial infarction (MI) within 72 h before enrolment; severe congestive heart failure (ejection fraction < 30%); prior surgery with the opening of the pericardium or pleura; patients with pleural adhesion; high risk or prior history of significant bleeding; contraindication to dual antiplatelet therapy (DAPT); combined other cardiac surgery; preoperative hemodynamic instability; oral anticoagulation therapy that cannot be withheld.

**Figure 1:**
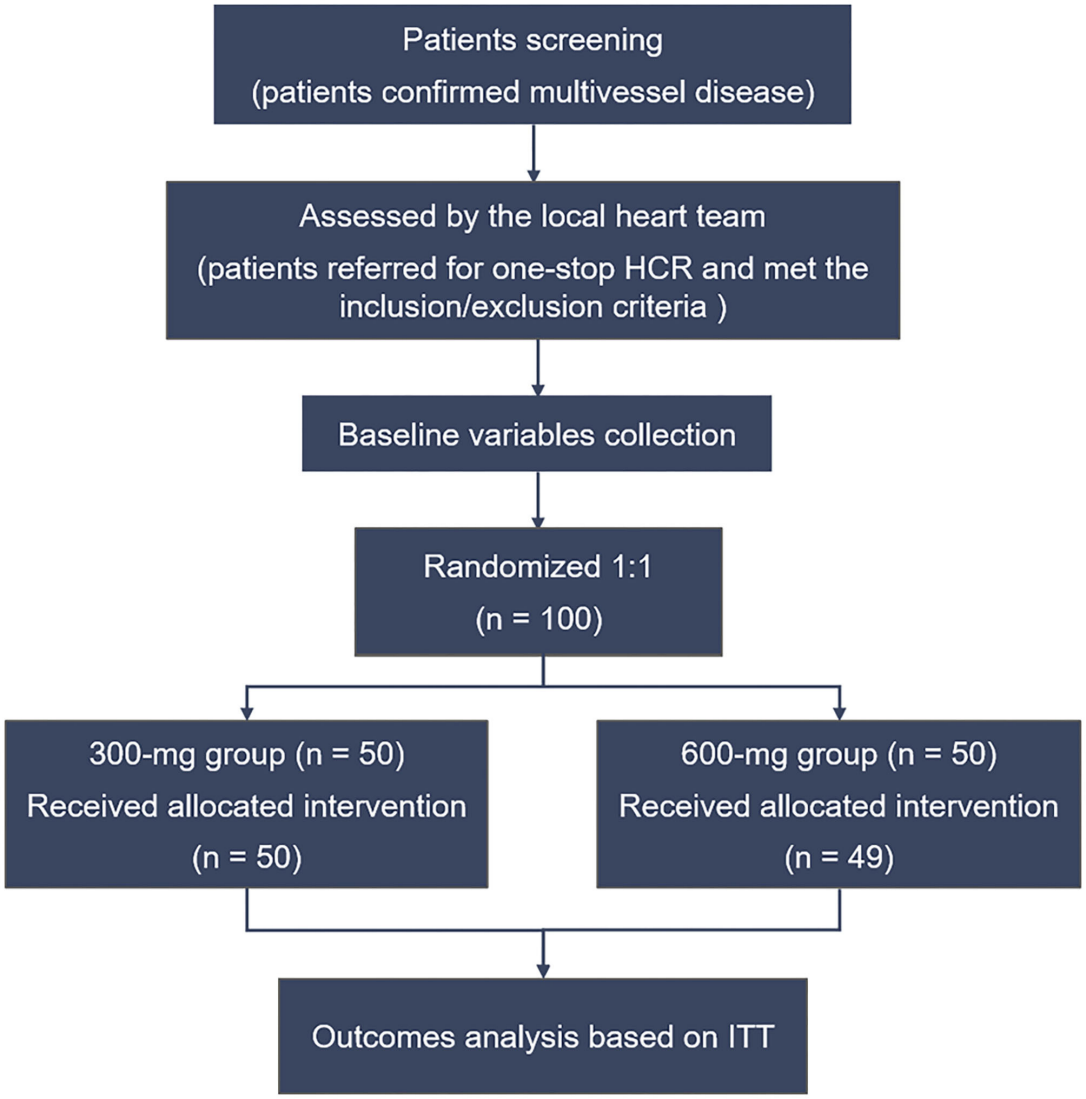
Flow chart of the study. HCR, hybrid coronary revascularization; ITT, intention-to-treat.

All the data were collected by the clinical research staff of Beijing Chaoyang Hospital (Beijing, China). The Case Report Forms were recorded and entered into the database by technicians. The enrolled patients were randomly assigned to the clopidogrel loading dose 300-mg group or 600-mg group in a 1:1 ratio before the HCR procedure. Randomization was blind to patients but not to operators and was performed according to the random table generated by SPSS software (IBM SPSS Statistics for Windows, Version 22). The first 50 patients were assigned to the 300-mg group, while the last 50 subjects were assigned to the 600-mg group. The study flow chart is shown in Figure 1.

The trial was conducted in accordance with the Declaration of Helsinki (as revised in 2013). The study was approved by the local research ethics board of Beijing Chaoyang Hospital (No.: 2018-D-186), and informed consent was taken from all individual participants.

### Interventions and Antiplatelet Strategy

Surgical and intervention procedures of one-stop HCR were described previously (9). Minimally invasive direct coronary artery bypass (MIDCAB) and PCI were performed in a hybrid operation room simultaneously.

Clopidogrel was discontinued 3–5 days before surgery, while aspirin (100 mg/day) was continued preoperatively. Unfractionated heparin was used to keep activated clotting time (ACT) > 300 s at the end of LIMA harvesting. After LIMA-LAD anastomosis, heparin reversal was achieved by a partial dose of protamine. Then, a loading dose of clopidogrel (300 or 600 mg according to randomization) was administered *via* a nasogastric tube before the closure of the thorax. ACT > 250 s was obtained by the second administration of unfractionated heparin in the PCI procedure. After surgery, all patients were treated with 100 mg aspirin within 6 h, then continued for life if there were no contraindications and combined with clopidogrel at a dose of 75 mg/day was administered for 12 months.

### Outcome Measurements

The primary endpoints were major bleeding events after the HCR procedure, including Bleeding Academic Research Consortium (BARC) type 4: CABG related bleeding (BARC CABG), PLATelet inhibition, and Patient Outcomes (PLATO) life-threatening bleeding, and PLATO major bleeding. These major bleeding events were defined according to four published studies as follows. First, BARC CABG may be perioperative intracranial bleeding within 48 h, reoperation after the closure of sternotomy for the purpose of controlling bleeding, transfusion of 5 U whole blood or packed red blood cells within a 48-h period, or chest tube output 2 L within a 24-h period (10). Second, PLATO life-threatening bleeding may be fatal bleeding, pericardial bleeding requiring repeat surgery, drop in hemoglobin of ≥ 50 g/L, or transfusion of 4 or more units of RBCs (11). Lastly, PLATO major bleeding may be pericardial bleeding requiring repeat surgery, drop in hemoglobin of ≥ 30 g/L, or transfusion of two or more units of RBCs (12).

The secondary endpoints were composite in-hospital ischemic thrombotic and adverse complications, including postoperative death, MI, new-onset ischemic stroke verified by CT or MRI, pulmonary embolism, or deep venous thrombosis during the index hospitalization.

### Statistical Analysis

Continuous variables normally distributed were expressed as *M* ± *SD*, while non-normally distributed data were shown as median (the 25th percentile and 75th percentile), and categorical data were summarized as a proportion. Comparisons of baseline characteristics and outcomes between the 300- and 600-mg groups were evaluated by *t*-test or Mann-Whitney U-test for continuous variables and the chi-square test or Fisher exact test for categorical variables. All analyses were based on the intention-to-treat (ITT) principle. All statistical data analysis was performed by SPSS (IBM Corp. Released 2013. IBM SPSS Statistics for Windows, Version 22, Armonk, NY: IBM Corp, USA). A two-sided *P* < 0.05 was considered statistically significant.

## Results

### Baseline Characteristics

From August 2018 to June 2021, 100 patients with confirmed MVD and referred for one-stop HCR were randomized to clopidogrel 300-mg group (*n* = 50) or 600-mg group (*n* = 50). One patient in the 600-mg group received 300 mg therapy. Based on the principle of ITT analysis, all patient data were recorded and analyzed according to randomization. The mean age was 64.2 ± 7.8 and 63.2 ± 10.3 in the 300- and 600-mg groups, and the majority of patients were men. There was a good balance between the two groups in demographic, clinical, angiographic characteristics, and baseline medication (Table 1). No patients conversed to sternotomy, and all patients received LIMA-LAD anastomosis. Intraoperative variables were not significantly different between the 300- and 600-mg groups (Table 2).

**Table 1:** Baseline characteristics of patients according to the study groups.

**Preoperative characteristics**	**300-mg group, *n* = 50**	**600-mg group, *n* = 50**	***P***
Age (years)	64.2 ± 7.8	63.2 ± 10.3	0.60
Male	76.0	74.0	0.82
BMI (kg/m^2^)	26.1 ± 3.0	26.0 ± 3.0	0.83
Hypertension	74.0	76.0	0.82
Hyperlipidemia	62.0	66.0	0.68
Diabetes mellitus	38.0	42.0	0.68
Peripheral vascular disease	8.0	2.0	0.36
Preoperative hemoglobin (g/L)	133.7	135.1	0.64
Preoperative creatinine (μmol/L)	72.0	69.4	0.45
Previous stroke	30.0	18.0	0.16
Previous MI	22.0	16.0	0.44
Previous PCI	26	26	1.00
Acute coronary syndrome	100	98.0	1.00
Left main disease	50.0	46.0	0.69
LVEF (%)	64.7 ± 8.3	64.6 ± 7.4	0.95
LVEDD (mm)	47.7 ± 3.9	47.6 ± 5.2	0.91
SYNTAX Score	29.4 ± 9.6	28.4 ± 7.9	0.59
EuroSCORE II	1.68 ± 1.08	1.60 ± 0.84	0.67
β blocker	68.0	74.0	0.51
ACEI/ARB	56.0	40.0	0.11
Statin	94.0	100	0.24

*Values are presented as mean ± SD or %. BMI, body mass index; MI, myocardial infarction; PCI, percutaneous coronary intervention; LVEF, left ventricular ejection fraction; LVEDD, left ventricular end-diastolic dimension; ACEI, angiotensin-converting enzyme inhibitor; ARB, angiotensin receptor blockers*.

**Table 2:** Intraoperative variables of patients according to the study groups.

**Intraoperative characteristics**	**300-mg group, *n* = 50**	**600-mg group, *n* = 50**	***P***
Conversion to sternotomy	0	0	NA
LIMA-LAD	100	100	NA
MGF (ml/min)	27.6 ± 16.8	22.8 ± 11.4	0.10
PI	2.4 ± 1.5	2.5 ± 1.2	0.86
Treated vessels	2.2 ± 1.3	2.4 ± 1.5	0.43
Number of DES/DCB			
1	32.0	26.0	0.51
2	40.0	42.0	0.84
3 or more	28.0	32.0	0.66

*Values are presented as mean ± SD or %. LIMA, left internal mammary artery; LAD, left anterior descending artery; MGF, mean graft flow; PI, pulsatility index; DES, drug-eluting stents; DCB, drug-coated balloon*.

### Major Bleeding

**Figure 2:**
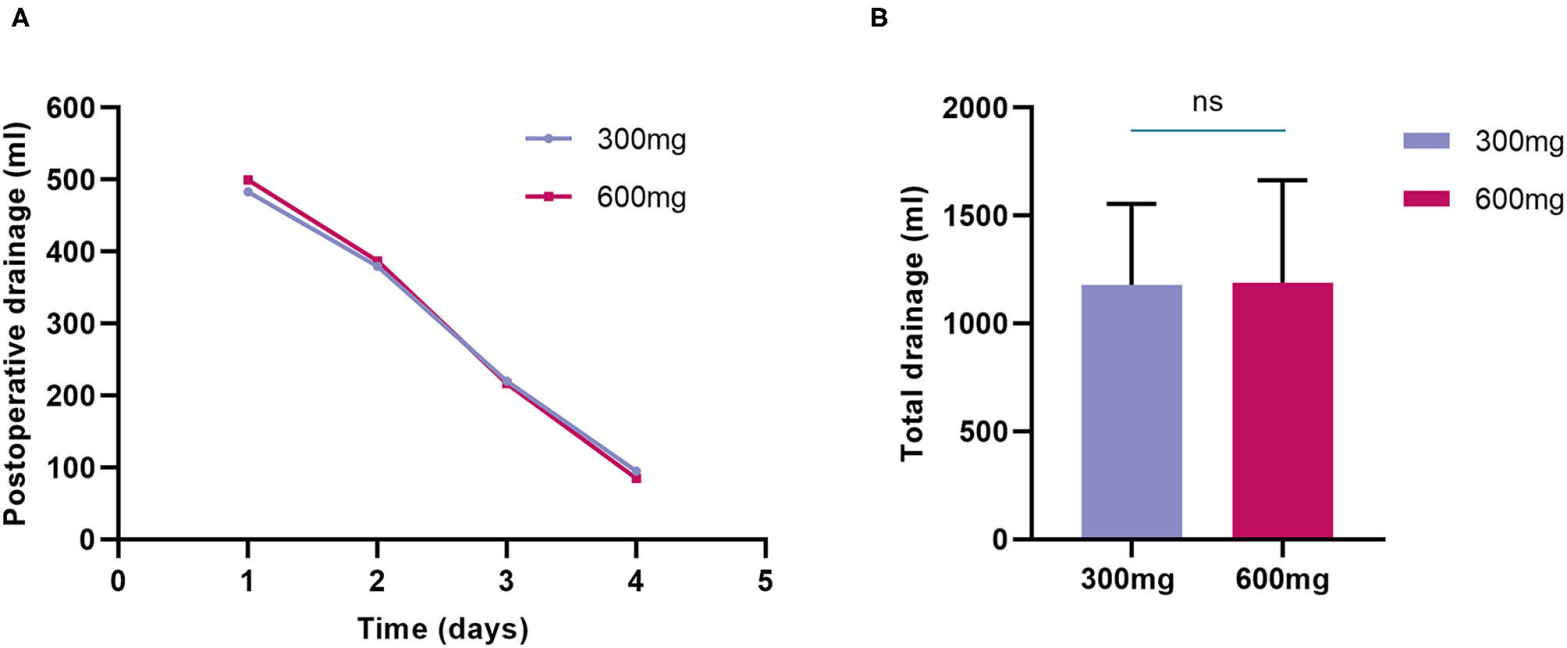
Postoperative drainage of randomized groups. **(A)** Postoperative mean chest drainage of the first 4 days was comparable between the two groups; **(B)** Total drainage volume was also similar between clopidogrel 300-mg group and 600-mg group.

Overall, the postoperative mean chest drainage of the first 4 days was comparable between the two groups (Figure 2A), and the total drainage volume was 1,179 ± 375 ml in the 300-mg group while 1,189 ± 476 ml in the 600-mg group (*P* = 0.9), respectively (Figure 2B). No significant difference was shown between the 2 groups in chest drainage (Table 3). Two patients in 300-mg group and one in 600-mg group received reoperation for bleeding (4 vs. 2%, *P* = 1.00). The proportion of postoperative transfusion and lowest postoperative hemoglobin were also similar.

**Table 3:** Postoperative outcomes of patients according to the study groups.

**Variables**	**300-mg group, *n* = 50**	**600-mg group, *n* = 50**	***P***
Postoperative drainage (ml)			
1st day	483 ± 219	499 ± 270	0.74
2nd day	379 ± 137	387 ± 178	0.82
3rd day	220 ± 144	217 ± 140	0.91
4th day	95 ± 124	85 ± 100	0.66
Total drainage	1179 ± 375	1189 ± 476	0.90
Reoperation for bleeding	4.0	2.0	1.00
Postoperative transfusion	22.0	18.0	0.62
Lowest postoperative hemoglobin (g/L)	96.7 ± 14.2	95.1 ± 16.9	0.61
Major bleeding			
BARC CABG	4.0	2.0	1.00
PLATO life-threatening	20.0	26.0	0.48
PLATO major bleeding	70.0	76.0	0.50
In-hospital death	0.0	2.0	1.00
Postoperative MI	4.0	2.0	1.00
Postoperative stroke	2.0	2.0	1.00
Thrombotic events	0.0	0.0	NA
Composite ischemic and adverse events	6.0	4.0	1.00
ICU stay (h)	142 ± 278	110 ± 93	0.43
LOS in hospital after HCR (day)	12.5 ± 5.9	11.1 ± 4.6	0.18

*Values are presented as mean ± SD or %. BARC, bleeding academic research consortium; CABG, coronary artery bypass grafting; PLATO, platelet inhibition and patient outcomes; MI, myocardial infarction; ICU, intensive care unit; LOS, length of stay; HCR, hybrid coronary revascularization*.

**Figure 3:**
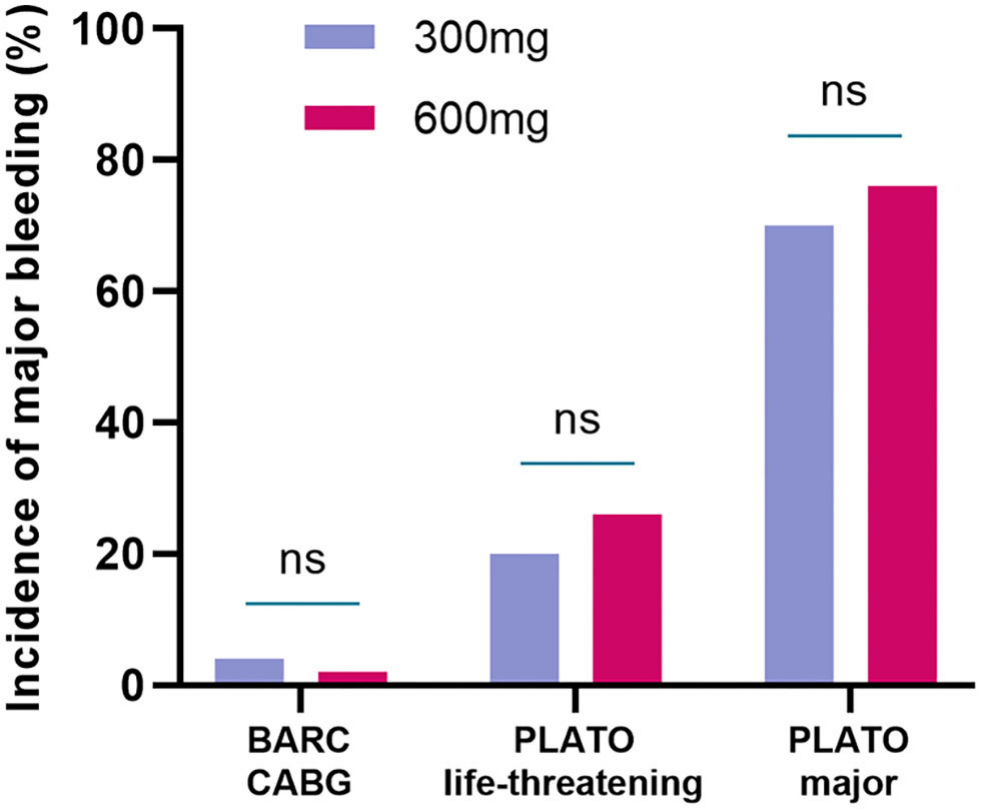
The incidence of major bleeding events in randomized groups. The incidence of BARC CABG, PLATO life-threatening bleeding, and PLATO major bleeding were not statistically significant between the 300-mg group and 600-mg group. BARC, bleeding academic research consortium; CABG, coronary artery bypass grafting; PLATO, platelet inhibition and patient outcomes.

For major bleeding events, no differences were found in BARC CABG (4 vs. 2%, *P* = 1) between the two groups. The incidence of PLATO life-threatening bleeding and PLATO major bleeding was slightly higher in the 600-mg group but was not statistically significant compared with the 300-mg group. PLATO life-threatening: (20 vs. 26%, *P* = 0.48), PLATO major bleeding: (70 vs. 76%, *P* = 0.50) (Table 3 and Figure 3).

### Secondary Endpoints

In-hospital death, postoperative MI, stroke, and thrombotic events did not differ significantly between the two groups. The composite ischemic thrombotic and adverse events were 6% in the 300-mg group and 4% in the 600-mg group and did not reach statistical significance (*P* = 1). Time of intensive care unit (ICU) stay and length of stay in hospital after HCR were also comparable between the two groups (Table 3).

## Discussion

Simultaneous HCR has several advantages compared with staged HCR. Firstly, complete revascularization in a single procedure, and avoiding of ischemia risk caused by untreated lesions in staged HCR. Then, when LIMA-LAD is finished, PCI to high-risk non-LAD lesions can be safer and easier. Thirdly, LIMA-LAD graft can be evaluated *via* angiography before PCI. Finally, condensing revascularization therapy in one patient encounter could make patients satisfied.

On the other hand, the potentially increased bleeding risk in the presence of clopidogrel in one-stop HCR may challenge the hemostatic technique of the surgeon and impair the outcomes, notwithstanding the less-invasive nature of the MIDCAB compared with off-pump coronary artery bypass (OPCAB) (5). In addition, the inflammation and abnormal activation of platelet function related to the surgical procedure would lead to perioperative thrombosis (13, 14). A balanced and optimized antithrombotic management is particularly significant.

The study of Panoulas et al. summarized the antiplatelet strategies of several studies, in most series of simultaneous HCR, patients are not premedicated with clopidogrel and undergo the MIDCAB taking only aspirin, followed by a single loading dose of clopidogrel 300 mg (7). Additionally, the study of Kiaii et al. adopted a loading dose of clopidogrel 600 mg in their procedures (15). In the latest randomized pilot study (MERGING trial), 40 staged HCR (PCI followed by MIDCAB) patients and 20 OPCAB patients were compared, the use of loading dose of clopidogrel was 600 mg in the staged HCR procedure, the major bleeding rates were 7.5% in the hybrid group and 5% in the OPCAB group (*P* > 0.99), and the acute stent thrombosis rate was 2.5% in HCR group (16). The HREVS trial is another randomized clinical trial compared HCR (*n* = 49), CABG (*n* = 49), and PCI (*n* = 51). In their staged HCR (PCI followed by MIDCAB) procedure, a loading dose of clopidogrel 300 mg was administrated at the time of PCI, the major bleeding (BARC 3–4) rate was 9.6% in the HCR group, and the myocardial ischemia rate was 5.8% (17). Thus, whether higher loading doses of clopidogrel (600 mg) would increase the risk of hemorrhage in one-stop HCR is an issue worthy of further investigation.

The current pilot study showed a comparable incidence of major bleeding between clopidogrel 300-mg and 600-mg group in patients undergoing one-stop HCR, which indicated the safety of clopidogrel loading dose 600 mg in one-stop HCR. However, our results did not show the advantage of clopidogrel 600 mg in composite ischemic thrombotic and adverse events (6 vs. 4%, *P* = 1). The likely explanation for this outcome lies in the low incidence of ischemic thrombotic and adverse events (3 patients in the 300-mg group and 2 patients in the 600-mg group) and the limited sample size of our study.

Nevertheless, the recommended loading dose of clopidogrel is 300–600 mg in patients undergoing PCI according to the recent European Society of Cardiology (ESC) guideline (18). Furthermore, increasing evidence has indicated that higher clopidogrel doses improve the antiplatelet effect and the outcome of the patient, which is due to the higher absolute level of platelet inhibition and more rapid onset of action (19, 20). A meta-analysis conducted by Siller et al. compared the efficacy and safety of two clopidogrel loading regimens (300 vs. 600 mg) in patients undergoing PCI, which demonstrated that a 600 mg clopidogrel loading was associated with a 34% relative risk reduction of major cardiovascular events defined as death, MI, stroke, and target vessel revascularization (TVR). Meanwhile, the 600 mg clopidogrel loading dose was not associated with an increased risk of major bleedings (20). Another cohort study analyzed 4,105 unselected patients who underwent PCI and were treated with clopidogrel loading doses of 600 and 300 mg. As a result, a 600 mg loading dose was associated with a significantly lower rate of major adverse cardiovascular events at 1 month and without any increase in bleeding complications.

Based on the above evidence, we hypothesized that administration with a higher clopidogrel loading dose (600 mg) in one-stop HCR could reduce the incidence of ischemic events as well, though our study did not demonstrate this benefit due to the lower incidence of these events and small sample size. Certainly, it needed more sufficient data to support this hypothesis in one-stop HCR.

There were several limitations to this study. Because of the pilot nature of this trial, although the safety of 600 mg loading dose has been indicated, it was not powered to detect the difference in effectiveness (composite in-hospital ischemic thrombotic and adverse complications). Furthermore, we did not test the platelet function before and after the treatment. The third limitation is that the proportion of clopidogrel resistance was unclear in this study because lack of selective gene profiling in clopidogrel patients, which may affect the study results. Finally, expanded sample size is required, but it was difficult to carry out due to the relatively high technical barriers and limited adoption of one-stop HCR.

## Conclusions

In patients receiving one-stop HCR, clopidogrel 600 mg loading dose did not increase major bleeding events compared with 300 mg. More sufficient data is necessary to evaluate the potential benefits of 600 mg loading dose in one-stop HCR.

## Data Availability Statement

The original contributions presented in the study are included in the article/supplementary material, further inquiries can be directed to the corresponding author/s.

## Ethics Statement

The studies involving human participants were reviewed and approved by Research Ethics Board of Beijing Chaoyang Hospital. The patients/participants provided their written informed consent to participate in this study.

## Author Contributions

YuG and DL: conception and design, data analysis, and interpretation. PS and YL: administrative support. JG, SG, XA, and XZ: provision of study materials or patients. DL, YiG, and JZ: collection and assembly of data. All authors manuscript writing and final approval of manuscript.

## Funding

This work was supported by Ministry of Science and Technology of the People's Republic of China Foundation (No. 2016YFC1301405).

## Conflict of Interest

The authors declare that the research was conducted in the absence of any commercial or financial relationships that could be construed as a potential conflict of interest.

## Publisher's Note

All claims expressed in this article are solely those of the authors and do not necessarily represent those of their affiliated organizations, or those of the publisher, the editors and the reviewers. Any product that may be evaluated in this article, or claim that may be made by its manufacturer, is not guaranteed or endorsed by the publisher.
